# What are the lived experiences of patients with cancer and their families in northern Ghana? A qualitative study using narrative interview and creative task approach

**DOI:** 10.1136/bmjopen-2024-093303

**Published:** 2025-02-26

**Authors:** Chloe Zabrina Tuck, Robert Akparibo, Laura A Gray, Hamza Suraj, Abdul-Rashid Timtoni Iddrisu, Tampuri Rahman Abane, Alhassan Ahmed Deedat, Richmond Nii Okai Aryeetey, Braimah Baba Abubakari, Amos Azure, Richard Cooper

**Affiliations:** 1 SCHARR, Division of Population Health, The University of Sheffield, Sheffield, UK; 2 Tamale Teaching Hospital, Tamale, Ghana; 3 Northern Regional Health Directorate - Ghana Health Service, Tamale, Ghana; 4 Population Family and Reproductive Health, University of Ghana, Accra, Ghana

**Keywords:** Adult oncology, Adult palliative care, QUALITATIVE RESEARCH

## Abstract

**Abstract:**

**Objectives:**

Cancer poses a major burden in Ghana that is exacerbated by poor engagement with biomedical treatment. The reasons for this are not well understood for most cancers and in northern Ghana.

**Design:**

This research took combined narrative interviews with a creative task that was analysed through reflexive thematic analysis.

**Setting:**

A tertiary treatment centre in northern Ghana.

**Participants:**

15 adult (>18 years) patients or their relatives who had been diagnosed and/or treated for cancer within the last 2 years.

**Results:**

The thematic analysis highlighted the psychological burden of cancer and ways participants cope and find meaning, including through religion, trust in biomedical treatment, and occupation and social support. The findings stress the negative impact of the financial burden, shame, worry and the spiralling poverty this causes.

The creative task was found to be resonant, emotive and more humanising, which is anticipated to be more effective when communicating with policy-makers and community members. The findings provide rich contextual insights to understand patients’ and relatives’ perspectives and frame their experiences within what was important to them.

**Conclusions:**

Together the research has identified a critical need for policy to consider the psychosocial, occupational, spiritual and financial needs of patients with cancer in northern Ghana. It has demonstrated narrative interviews with graphical elicitation as an effective approach to discuss sensitive topics for findings that can engage stakeholders and inform holistic cancer service design.

**STRENGTHS AND LIMITATIONS OF THIS STUDY**

- The narrative interview approach provided patients and their relatives an opportunity to freely share their cancer stories.
- This was combined with a creative task which added unique insights into how patients interpreted their experiences and what they valued most.
- The approach was reflexive, working across cultures and considering how future work can seek to redress power asymmetries.
- The interviews required interpretation and translation to English, which may impact how the accounts are viewed and perceived.
- Due to poor outcomes, not all patients with cancer voices are included in the sample.

## Introduction

 Cancer poses a huge burden on health and well-being globally, which is increasing in many low-income and middle-income settings, including Ghana. According to Global Health Observatory estimates, there were 15 802 cancer-related deaths in 2020 (based on figures released in 2022), with liver, breast, cervical and prostate cancers contributing the greatest burdens.^1 2^ By 2024, this number had risen to 17 944, with liver cancer remaining the leading cause of death.^3^ While the prominence of breast and cervical cancers is increasing, many other cancers receive relatively less attention, particularly in terms of policy prioritisation, as highlighted and discussed in our previous literature reviews.^2 4^

Additionally, multiple social, cultural, health systems and environmental factors have been found to influence how patients engage with cancer care.^2 4^ For example, gender norms influence women’s decisions around mastectomy for breast cancer.^5^ Patients delay medical engagement due to first seeking traditional medicines respected in their local community or the cost of medical treatment.^5 6^ Delayed engagement can mean patients do not receive timely access to services, leading to worse outcomes.

Previous research has highlighted barriers to engaging with cancer services across the patient pathway in Ghana, from reaching facilities, navigating services, to accepting and completing treatment^4^ spanning all levels of the social ecological framework.^4^ Findings from our earlier review of the literature found that although there was a substantial amount of literature on breast and cervical cancer, there was a dearth of information on what influenced treatment completion for other cancers.^4^ The review uncovered that as studies were centred in southern Ghana, less was known about northern Ghana,^4^ where differences in socioeconomic status and culture may influence treatment behaviours and beliefs.^7^ In a published cross-sectional analysis from an earlier stage of this research, we identified treatment incompletion as a significant concern in northern Ghana.^8^ The cross-sectional study found missing data for all types of cancers, with particularly large gaps for certain cancers other than breast and cervical cancer.^8^ Moreover, the reasons for high drop-out were unknown. Therefore, in the current study, we aimed to explore the reasons for non-completion in this setting using a qualitative approach. Thus, the research question addressed was: What are the individual stories of adults with cancers and how do they interact with services in northern Ghana?

## Methods

This qualitative study was conducted as part of a larger mixed-methods study aimed at exploring the factors influencing cancer treatment uptake and identifying strategies to improve treatment uptake in Ghana.

The research methodology was situated within the social justice paradigm.^9^ This paradigm accommodates various forms of evidence, recognising the social, cultural and historic contexts that influence the priority given to each.^9 10^ Different types of evidence play distinct roles in driving social and political change. This study applied qualitative methods to uncover and provide a rich contextual understanding of the social experiences of living with cancer.

The qualitative methodology used allowed us to explore social phenomena such as treatment acceptance and associated patient experiences and beliefs, with an emphasis on recognising multiple worldviews and the social construction of these. Acknowledging the social nature of experience and striving to not impose externally defined frames relating to cancer experiences, this study applies a method that privileges the participants’ views and priorities. One such method is narrative interviewing, which allows participants to tell their story without a predefined agenda.^11^,^13^

It is important that any interview approach is sensitive to the participants’ needs, as cancer can bring with it many strong emotions which may also be difficult to articulate. Moreover, the researcher–participant power dynamic can be daunting.^14^ Approaches such as art therapy can help patients make sense of their condition and express inner experiences that they may struggle to put into words, in a more relaxed environment.^15^ Recognising the limitations of language to articulate experiences, together with the power dynamics between the researcher and participant and sensitivity needed with cancer, cancer narrative interviews have been built on to include creative methods such as art elicitation tasks (graphic elicitation technique).^16^ Graphical elicitation is an established technique^17^,^19^ which was considered suitable for research with patients in Ghana as it is known to be helpful in cross-cultural settings and when language is limiting.^20^ This can involve a variety of different visual art forms, including collage which may be particularly relevant as an art form that can be empowering but requires no prior skills, unlike drawing.^21^ Using both collage and interview methods together can bring greater focus to salient points of a patient’s condition and reveal unarticulated experiences such as mental states, hopes and coping skills, facilitating deeper reflection on mood and emotions.^21^

It is important that creative methods are context-informed.^15 20^ Cross-sectional data analysed at another stage of this research study suggested 75% of patients treated for cancer have not attended any school, thus some, not all participants may feel comfortable writing or drawing. Given the rich culture of fabric work in Ghana, incorporating fabrics may offer an approach participants feel comfortable with. In the art therapy discipline, use of fabrics has been found to help personal expression, improve communication and meditative skills.^15^

The approach taken here combined a narrative interview with a creative task using fabrics. We provide a critical reflection on the approach later in the discussion.

### Participant and recruitment

15 adult (>18 years) patients who had been diagnosed and/or treated for cancer at a teaching hospital in the north of Ghana or the relatives of such patients with cancer, treated within the last 2 years, were purposively sampled. They were from the five northern regions (Northern, Northeast, Savanna, and Upper East and West regions). The purposive sampling used sought to cover a broad range of social experiences and maximise variation in location, age, socioeconomic status and cancer types, which we found to be lacking in existing literature.^4^ Sampling was stopped after 15 participants as theoretical saturation was reached. No participants who were invited declined or dropped out of the study. Initially, we planned to interview patients only; however, previous research has found that for some cancers, where less is known, survival rates were very low.^2 22 23^ For liver cancer, the patient population is described as having no voice. Thus, excluding patient relatives when patients are not available could lead to bias. Patient relatives were thus included in the study, especially when patients were not available. This followed the same inclusion parameters as patients; however, instead of being a patient treated at the site within 2 years (over 18 years), they were individuals who had regularly accompanied a patient for treatment during that time frame. Participants were invited and sensitised to the research by a local nurse with whom they already had contact with, using the information in the participant information sheet. After agreeing to participate in the interview, a time and location were agreed based on the participants’ preference. This was intended to ensure they felt comfortable to speak. Recruitment occurred between 17 July 2023 and 28 August 2023.

### Data collection

Data collection took place at the hospital’s Oncology unit, from July to October 2023, using an interview guide (see online supplemental information). Interviews were conducted in the preferred language of the participant and included: English, Dagbani, Kusaal or Twi. The lead author (CZT) led the interview in English, which was translated to other languages by a preselected interpreter (qualified male nurses with oncology experience, HS/AA). The interview guide was made flexible to accommodate participants’ reflections, questions, as well as it allowed the interviewer to clarify doubts. It included an introduction which explained the aims of the research and the lead researchers’ reasons for pursuing the study. After the interview questions, participants were invited to take part in a creative task. The aim of this was to use a piece of fabric framed by an embroidery loop to communicate any message they would like to share about their experience. This could be done in whichever medium they preferred or a mixture, using pens, paints, fabrics, thread or beads. After the task, they were given the opportunity to provide any additional feedback. The interview process and approximate timings were explained at the outset. The respondent was assured that there was no right or wrong way to perform the task, and that they were the expert on their experiences. After the interview, the participant was followed up by an interpreter thanking them for their participation, checking they returned home safely and ensuring that the interview had not caused any distress. Along with the interview transcripts and fabric collage artefacts, the lead author kept journal notes to reflect on the interviews. On average, the interview and creative process took 1 hour to complete.

### Data analysis

Interviews were transcribed verbatim by three transcribers (postgraduate students experienced in transcribing and briefed on the research and task) and validated by the lead author (CZT). Where required, translation was conducted by the authorship team and discussed with them to resolve discrepancies. The transcripts with the notes and artefacts were analysed together using the process of reflexive thematic analysis (RTA).^24 25^ This involves six stages: first familiarisation with the data, followed by initial coding. The codes are then grouped together to form initial themes, which are reviewed and reassessed. The themes are refined, and names are assigned. Finally, each theme is articulated in the write-up. This analysis was conducted manually to allow greater immersion in the text, using multiple colour highlights and mapping. This process is theoretically flexible to align with a participatory approach. The RTA was adapted, drawing on work that considers the contextual position in which codes appear,^26^ through overreading and recontextualisation of themes within cases.

Analysis drew together evidence, identifying similarities and divergences. Themes were identified from the transcripts inductively, rather than being guided by existing frameworks. This decision was taken as frameworks may overlook some evidence if they take perspectives exclusively from the global north.^27^ This considered semantic insights (at the surface level) and latent themes (that go beyond, involving interpretation of underpinning meaning assigned to the data).^28^ The approach involved a non-linear process, recursive, requiring movement back and forth between phases, and reflecting on new findings and developing the approach taken appropriately.^28^ Initial codes were generated by CZT and reviewed either by RA and RC. Any discordance in codes was resolved through discussion. CZT also developed the initial themes which were reviewed and discussed with RA, RC and LAG. It was not feasible to share the transcripts and findings with participants due to low literacy rates. However, a graphical booklet is being developed alongside this research, which incorporates participants’ feedback.

Informed consent was obtained for all participants. A participant information sheet was explained in their local language. Consent was documented through signature or thumbprint, which was witnessed by the interviewer, interpreter and an independent witness and was securely archived.

There is a need for additional sensitivity in using graphical elicitation with patients.^21^ This was considered using several approaches: the draft guide was reviewed by an art therapist and oncology nurses at the hospital to ensure that it was sensitive to patients. Second, although the lead author (CZT—who led the interviews and data analysis) was from outside of Ghana, they spent several months volunteering at the hospital’s oncology centre in the northern region of Ghana where this research was carried out. This allowed CZT to familiarise herself with the patients’ needs. CZT also worked with local interpreters who had built rapport and trust with the participants. They also continually held debriefing sessions after each interview with participants to discuss the impact the process might have had on them, and how interaction could be improved.

### Trustworthiness

This approach incorporates several features anticipated to create trustworthiness in qualitative research—credibility, transferability, dependability and confirmability.^29 30^ Credibility relates to the research as an authentic representation of reality, which was achieved through peer debriefing, thematic saturation and triangulation of results. After each interview, the lead author held debriefed meetings with the interpreters to highlight strengths, limitations and where improvements in the approach could be made and minimise bias (confirmability). Dependability was created through transcription, meeting notes and an audit trail. Qualitative research seeks transferability, rather than external validity, which was sought through rich description and combining both speech and visual evidence to provide greater context.

### Positionality and reflexivity

A researcher’s subjectivity is inherent in RTA. Although the lead researcher (who collected and analysed the data) was from outside of northern Ghana, she lived and volunteered in the setting and worked closely with healthcare professionals to improve their understanding of the local context. During data collection, CZT (a white female researcher), who led the interviews, acknowledged the complexities of what her identity may bring to the research, particularly when interviewing non-white participants. She was aware that her position is shaped by her cultural background, upbringing and experiences, which may differ significantly from those of the participants, whose lived experiences are informed by different cultural, racial and social contexts. It is also important to recognise how these differences may influence the ways in which she engages with the participants, interprets their responses and makes sense of their experiences during analysis of the data. CZT was sensitised to academic and literacy writing on colonialism and positionality, to establish a stronger, critical acknowledgement of the colonial legacies that have shaped international research and reflect on how that may impact how they are perceived.

While CZT acknowledges her subjective interpretations are positioned as an outsider, it was felt this also lent to broadening perspectives of the findings.

## Findings

15 interviews were conducted with patients and/or their relatives. Eight interviews were with patients alone, five patients also had relatives present, while two were with relatives only. Of the five interviews with patients and relatives, most involved joint participation of the patient with their relative, except one patient who allowed their relative to solely partake in the interview and creative task on their behalf. On one occasion, a relative stepped in to partake in the creative task only. The patient pool is summarised in table 1.

**Table 1:** Summary of participant characteristics

Pseudo names	Position	Cancer type	Sex
Wumpagli	Patient with relative present	Breast	Female
Nabia	Patient	Gluteal mass	Male
Boresa	Survivor	Breast	Female
Tuurosung	Survivor	Sarcoma	Female
Zooya	Patient	Osteosarcoma	Male
Tipagya	Patient with relative present	Ovarian	Female
Beteyang	Patient with relative present	Gastric	Male
Napaga	Patient	Ovarian	Female
Banbio	Patient with relative present	Sarcoma	Male
Alamisi	Survivor	Breast	Female
Maakufaba	Patient	Breast	Female
Bakpama	Patient with relative present	Head and neck	Male
Naazo	Patient with relative present	Head and neck	Male
Salifu	Relative	Liver	Male
Asibit	Relative	Liver	Male

### Summary of study findings

**Figure 1:**
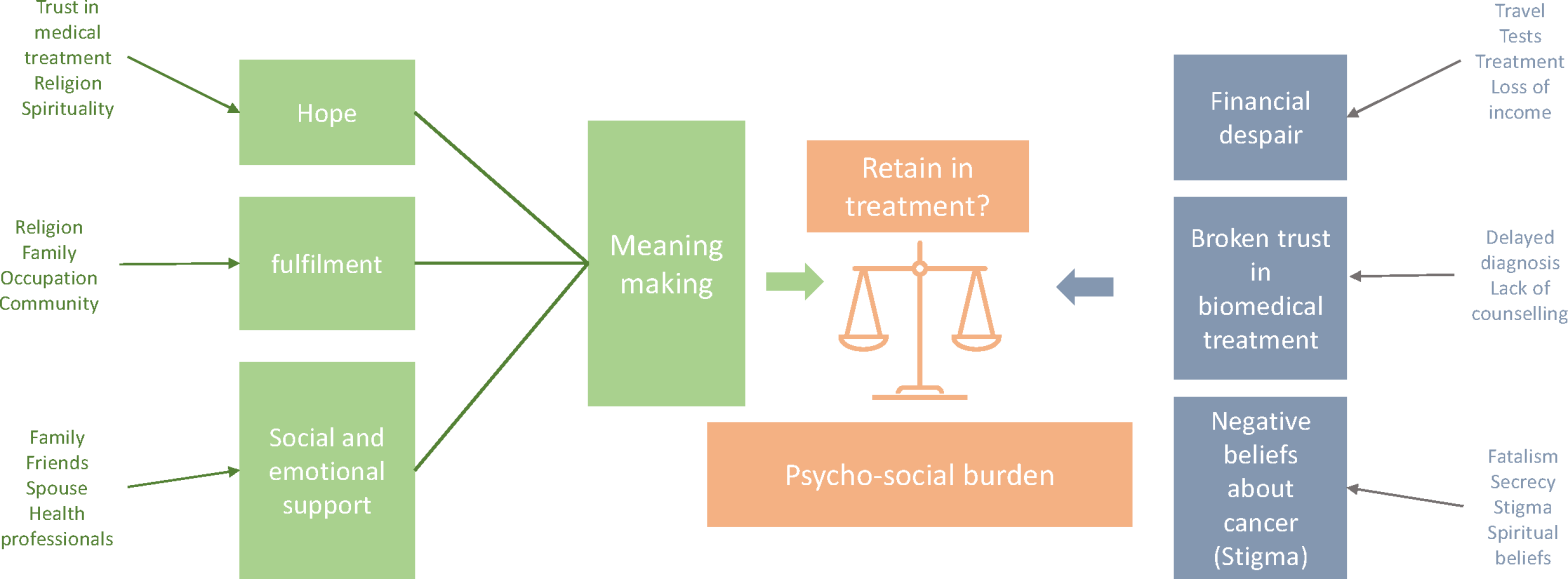
Main themes identified through reflexive thematic analysis. Elements in green helped patients cognise their condition, relieve psychosocial burden and stay in treatment. Conversely, themes depicted in blue were inhibitory.

The narrative interviews and creative task indicated the psychological impact of cancer and the lasting impact this has on participants. There were several ways patients and relatives were able to make sense of this, find purpose and cope with their diagnosis. Key among this was spirituality and religion. This was also supported by medical counselling and the faith patients had in treatment after seeing positive results. One factor that caused the greatest mental impact was the worry associated with financing their treatment. Lack of funds caused poverty in several households and led some patients to delay treatment. Several patients felt like a burden on their family, while relatives spoke of the psychological burden the diagnosis had on them. The findings are displayed graphically in figure 1 and further presented thematically below.

### Mental health burden of cancer

There was widespread mention of the mental health burden of cancer. The mental health burden concerned all stages of the treatment process—diagnosis, deciding on and pursuing treatment.

Boresa spoke about crying and being very nervous before their diagnosis as there was a belief that cancer was a death sentence. This was echoed by Banbio and Beteyang, who initially believed they would die of cancer.

Many participants also spoke about the worry and distress caused by the financial burden of cancer, and feeling like a burden on family members as they are not able to work. This led two participants to consider suicide at points in the treatment process.

**Figure 2:**
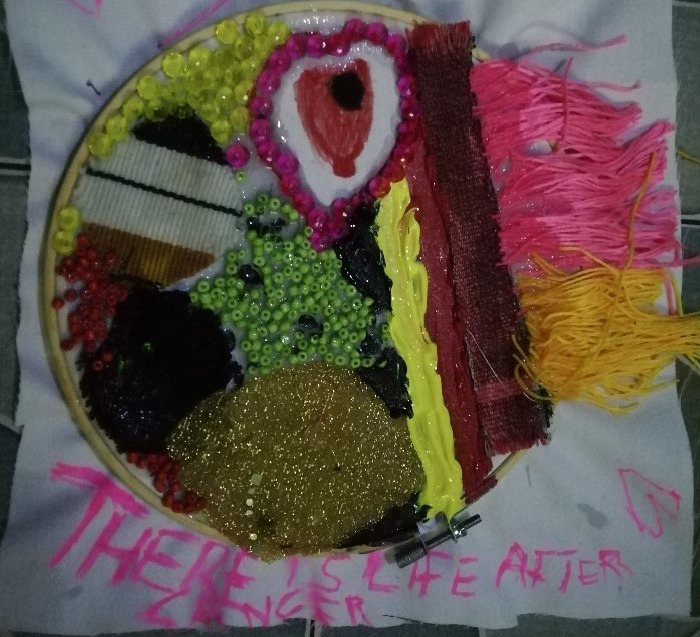
‘There is life after cancer’. Boresa, after recovering from breast cancer, spoke of the mental burden of cancer and used the creative task to highlight the struggles and the need for psychosocial support. *“[I] drew a heart so I want to place the beads, the pink beads on the heart…. to let the world know that breast cancer patients really needs love in Ghana. … …. there will soon be darker days even after my breast cancer journey, even though life is going to be blossom like the way these beads are green, but there are days I will still be down …. So that is why I have a little bit of black in between the green and then the hope is more than the doom. You see the black is small. The yellow stands for the hope, the red stands for my fight, the way I was able to come out of it, fight it.”*

The psychological burden was often illustrated graphically as a dark colour. Participants used the creative task to stress the darkness and challenges with cancer, often using the black colour, as illustrated in figures2^4^. Boresa spoke about darkness, even after completing treatment and contrasted it to colours symbolising new hopes and ambitions. Although she had completed treatment, she spoke about the lingering mental health toll years after, that some days are still difficult.

**Figure 3:**
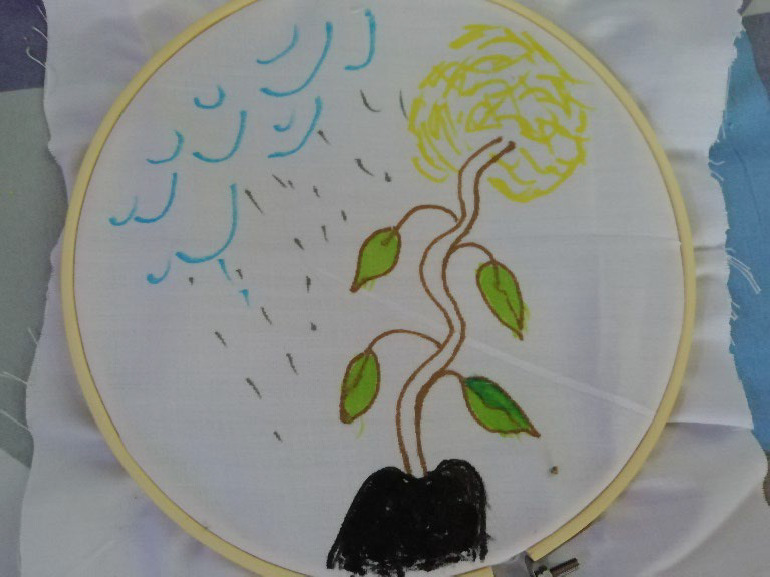
‘Cancer is real, but there is hope’. Tuurosung uses a plant metaphor to shed light on how she felt during her treatment journey. *“the base of the tree is black…… felt that it was dark for me everything was just dark……there was no way. I was devastated but when I look up to the sky, the sky was seen blue and then I felt in my heart that there is hope for me. The doctors the medical team told me there was medication for it, for the cancer, so I hope and then rain came down for me, so when the rain came down for me….You know when the rain was coming direct on me that is the chemo drugs [that] they were giving me. Everything was on me and then friends encouragement, family encouragement, all those things sum up to the rain that was pouring on me … that’s why I use the droplet here…then the green leaf indicating a leaf shooting up a new beginning for me though I was broken and rain came down on me. People will pray for me …. encouragement so I had hope…”*

**Figure 4:**
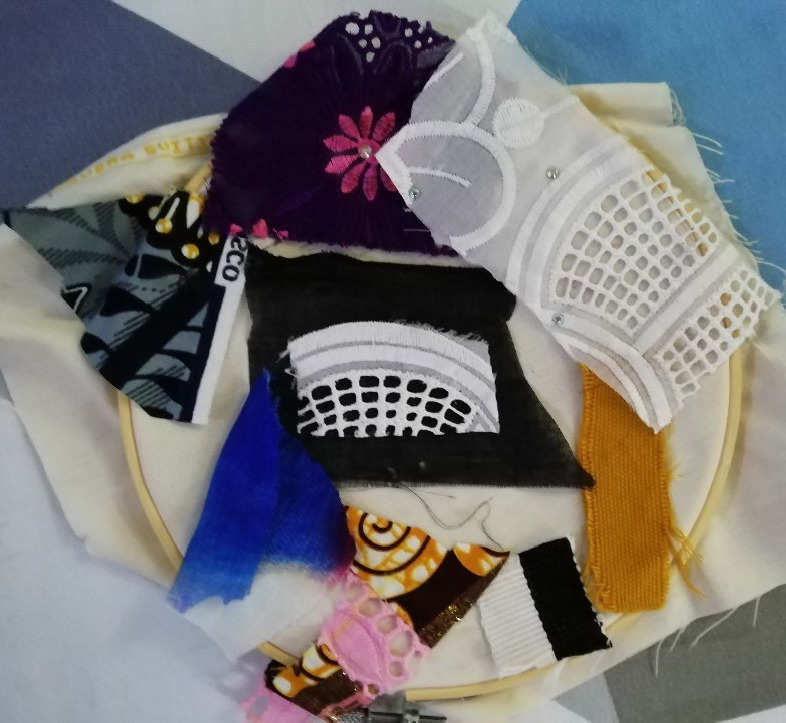
Untitled participant artwork. Beteyang shares his interpretation and way of coping. "*I am the one here, in the middle [white rectangular shaped piece of rag] after I was diagnosed with the sickness and I was in a deep darkness [black rag] not knowing how to tackle the situation. The rest of the rags with different colors are those family members and health workers who are surrounding me, giving me advice and hope in different capacities to strengthen me. It shows that when you are diagnosed of cancer you don't have to throw yourself in despair, and also, family members shouldn't abandon you because you will be a burden to them. If the family inspires you with hope, you will get healthy again*.”

### Mental burden on relatives

The emotional burden was also felt by relatives; the relative of Tipagya described herself as ‘emotionally sick’, while indicating Tipagya was not disclosing all their symptoms to not distress them. While also being distressed at her own diagnosis, Alamisi also understood the burden on relatives after being distressed after her mother’s death from cancer. She indicated she felt depressed for several years after her mother’s death and would not wish that on her own family.

Asibit spoke about the financial worry and suffering of supporting a liver cancer patient. This felt tiring yet hopeless. He spoke about how it caused him to struggle eating and sleeping due to the psychological impact.

Salifu, on the other hand, said he was psychologically impacted by his father’s condition. He felt the situation was hopeless and felt worried and alone as he stayed at the hospital with him. The brother of Banbio also felt a victim due to the mental burden on him from the news.

### Learning through the mental health burden of cancer

For several patients, the mental health burden of cancer was made worse by other life events, such as divorce, supporting an estranged family and chieftaincy disputes. Zooya had been supporting his estranged family, undergoing a divorce, feeling alone, co-habiting with strangers, while also being treated for cancer. However, Zooya was able to see these experiences as part of a journey and advised others to keep positive. He said he felt more caring and empathetic to others, as he could relate to what they were going through after these experiences. Similarly, Nabia felt they were not fazed by the diagnosis and were able to see the positives in any situation. Maakufaba felt the diagnosis had made her a more pious, calmer person and given her an opportunity to learn of new places.

### Finding purpose through occupation

For several participants work and study gave them hope for the future and a sense of purpose. Zooya talked about creating a sanctuary in his room where he could work from home: *“I made it so cozy and it was so sanctuary when I go in there I don’t want to come out, I just stay in the room it is my little world because that time I had created my accounting app and I was doing my online business …”*

Nabia said he felt well supported by his employer who supported with costs. However, other patients struggled with fitting in at work, despite the positive mental health support work offered. Tuurosung spoke about being tired and having back aches at work and feeling the need to hide side effects of treatment, such as having blackened hands, from customers. Tuurosung said they were not able to study whilst undergoing treatment, but this was a motivation to recover. Similarly, the relative of Tipagya spoke of the patient’s hopes to study and register at a health profession training school. Contrasting this, at times they also lost hope due to their uncertainty. The relative paraphrased *“Do I have a future?”*.

**Figure 5:**
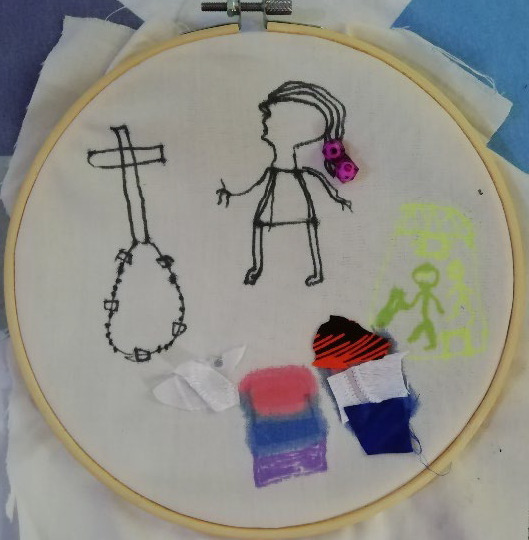
Untitled participant artwork. *Alamisi,* spoke about what helped her recover from breast cancer. “*This is me (name) a cancer patient who survived, this is my cross, the Lord that makes me alive when I was sick, and this white means all my family that make their heart clean to help me when I was going through the pain. … And this symbol here is my working place, those who helped me and still come to my place some use red, blue and violet colour doing their hair, … and pay me so that I get enough money to go to hospital…. And this one too is the NPP members. They are the one when I was suffering also took part on my health and also help me with their amount of four thousand for the hospital. And this one too here is the hospital that we are going, the doctors there, the nurses there. They used their brain and their time to take care of us and make us well ‘til I’ve finished all the chemo and the treatment and now I am fine.”*

The value patients took from work and motivation to recover through it was also highlighted in the creative task. Alamisi drew themselves working in the service sector and regaining their occupational status after illness (figure 5). They used beads that they commonly put in hair to empathise this.

Patients struggled with not being able to work, which made many participants lose a sense of worth, commonly talking about being stuck in their house while their family supported them.

Bakpama’s treatment journey intersected with a time when they lost work due to chieftaincy disputes in their village. This left them relying on others for food and lodging, leading to them feeling helpless.

Loss of purpose was exacerbated by patients struggling with day-to-day activities and needing support from family. Tipagya spoke that they would be better off dead due to the distress they are causing their family. *“So I sent my husband a message on WhatsApp and I told him …I can see I have become like a burden on him so I think taking my life will be the best for him…”* – Boresa

### Finding purpose through family

Another factor that gave participants hope was their family. Boresa talked about trying to conceive and hoping to have children in the future. She saw looking after her children as her role and worried who would look after her children if she were to die. This gave a sense of purpose to continue.

Alamisi said she was motivated to continue treatment and survive as she did not want her children to suffer if she died. She spoke about having a mastectomy, despite knowing that she would lose a breast and the stigma this may cause, as she wanted to live to take care of her children:

*“I want my life and to be there taking care of my children.”* The impact of cancer on family dynamics was depicted graphically by Salifu in online supplemental figure 1).

Family brought a sense of purpose and peace of mind. This was also articulated in the creative task, Naazo chose traditional smock fabrics which they identified with to illustrate themselves at peace with God and their family (online supplemental figure 2).

### Finding purpose through serving the community

Zooya spoke about how the experience of cancer had made him more caring and empathetic. This led him to support other patients who were struggling financially by paying for their medicines and travel. He donated equipment to the oncology department to support their work. He inferenced that wealth has no value without community benefit.

[…] my question is if you have the wealth and the community doesn’t benefit from it for me it’s not a good wealth … For me even the satisfaction you will get from changing people’s lives right is immensely you can’t put any price on it.

Boresa, who identified as a survivor, spoke about wanting to help others and initiated a support network to give a platform for patients with cancer in the northern regions to share and learn. This was after support received from a similar network for breast cancer survivors in the south of the country who meet up to do occupational activities, such as cooking, dancing and games. She felt this was lacking for patients in the northern regions and wanted to start this.

### Religion

A dominating theme in how patients made sense and were able to cope with the many stresses that came with a cancer diagnosis was through religion.

Wumpagli emphasised how grateful he was to Allah (God) for their gradual healing. They said, *“what we want is what God wants too”* and had hope *“God will intervene”*.

Similarly, Tuurosung and Napaga spoke of the trust they had in God, and hope came from religion for Boresa. Bakpama also had trust in God to help with their psychological burden. Naazo found their diagnosis scary but they gave everything to God and prayed for God to direct them to someone to cure them.

For Maakufaba, having faith had been pivotal in coping with their diagnosis. Initially they were not very religious, but it was a key coping mechanism for their bad dreams and thoughts of death. Praying also helped them cope with the worry of how to finance their treatment.

The relative of Tipagya consoled her to have faith in God. Tipagya prayed as a Muslim. Her relative, as a Christian, found religion was key for her to cope. She went to a church program where members came together to give testimonies and pray for each other. She highlighted religious coping in her creative task (online supplemental figure 3). Similarly, Salifu used religion to help cope with the hopelessness he felt while her father was unwell with liver cancer.

**Figure 6:**
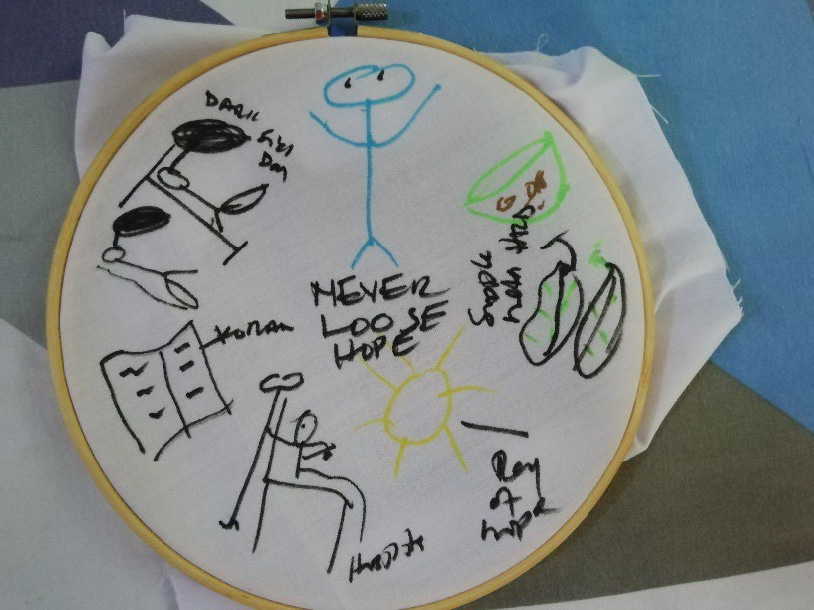
‘Never lose hope’. Zooya, uses the creative task to share several different elements that they believe are helping them overcome cancer. *“… sometimes lying on the bed, I lie on the floor, I hardly can sit and this is the Quran. I was listening to, and when I come to the hospital I always [have] some ray of hope there all the time because I know… I am going to be healed … just the journey so that is the ray of hope and this is a hospital to get a treatment. …anytime I come to the hospital I try to be positive that I’m being healed. Yeah, because I’ve seen the medication is working. …when I come back from the hospital I apply the supplementary which is the herbs and then at the end of the day there is a hope that I will be healed. …that’s me here it’s a bright colour and this is also green here, this is the herbs and everything, this is the leaves, this is the calabash, I put the herbs into the calabash and them I apply…”*

In their creative task and discussion Zooya spoke about how reciting the Quran helped her cope (figure 6), and how friends were praying for her.

**Figure 7:**
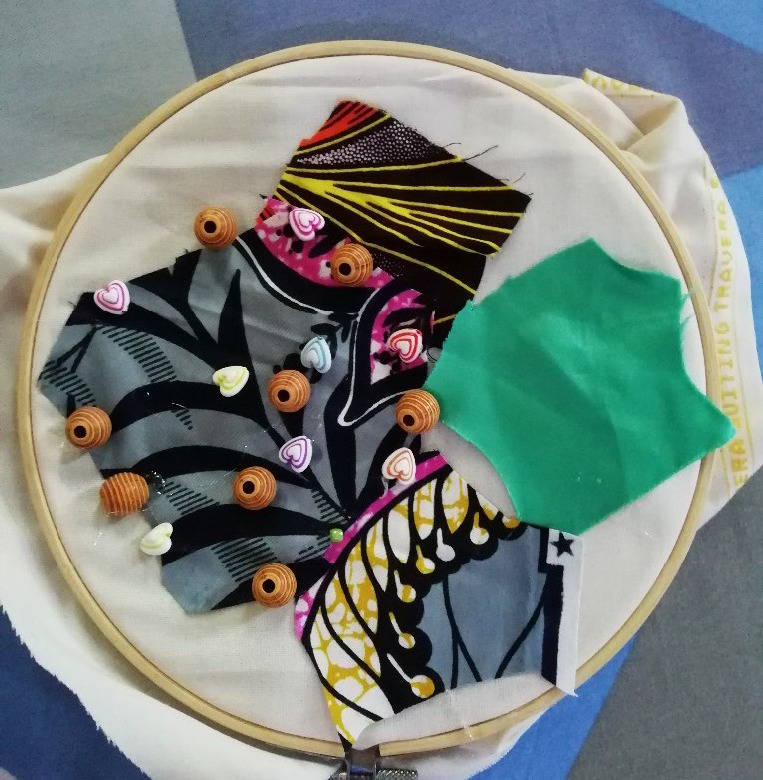
Untitled. Maakufaba shares a message about how she has found meaning. "This bead [white with green strips] is my pastor standing very close behind me and praying for me [brown colored bead] in the middle. The other two closer beads are junior pastors, and the rest of the brown beads are members in my church, and we are all standing together, and they are praying for me and supporting me in different ways, signifying that what has befallen upon me, I have not been abandoned by them.” "The rag here is also showing that you [individuals] should not leave yourself 'naked', meaning don't earn income today and spend all, save a little and use it one day as a piece of cloth to cover your nakedness, because this sickness [cancer] is a disgraceful sickness…. Then there will be new life [green rag] come at the end of the day”

Another way in which religion helped was through the support of their religious community. Alamisi, who was a catholic felt supported by prayers from her father and congregation, who also encouraged her chemotherapy. Similarly, Maakufaba spoke of the congregation praying for her and giving small amounts of financial assistance for her travel for treatment. She depicted this in the creative task using beads to represent herself, the pastor and junior pastors praying for her (figure 7).

Common among the participants was the fluidity of religion and accepting prayers and religious treatments from other religions. Nabia, a Muslim, but spoke about support from a friend who was a pastor.

### Traditional modes of treatment

Traditional and spiritual treatments were suggested by Nabia’s family who wanted him to seek a local solution. Boresa also indicated that her family brought local treatments, but her husband stood his ground and she instead pursued biomedical treatment. She suggested other patients were using herbal treatments out of desperation and to avoid mastectomy, as their husbands may remarry. Tipagya’s relative indicated she had received Qur’anic prayers and an ointment for the patient, suggested by other family members.

For several participants they first sought herbal treatments, Alamisi thought her breast lump was a boil. Bakpama’s head and neck cancer initially started with a tooth ache and swelling, which he treated with herbs as he feared going to the hospital until it got worse. For Naazo and Wumpagli, they lost faith in local treatments after experiencing their inefficacy. Wumpagli first sought local treatments due to the delay and time required to renew her National Health Insurance Scheme (NHIS) status. Relatives of patients with liver cancer spoke about how the patient first sought ineffective herbal treatments. Asibit expressed frustration as the patient took herbal treatments costing 2000 Ghana Health Service and leading to a month delay. Salifu spoke about how her father first sought herbal medicines, some provided by extended family members. After being referred to the teaching hospital, his father again opted for local treatments which Salifu perceived to be ineffective. It required forceful family intervention to bring the patient to this hospital, but there were further delays, as by this time the referral letter had expired.

The use of traditional spiritual and herbal treatments was well articulated by Zooya. He took a pragmatic approach to concurrent biomedical and local treatment. He spoke about not discarding local treatment but warned that other patients choosing this first could lead to delayed diagnosis. He suggested that his condition had a physical and spiritual manifestation that required both biomedical and spiritual intervention.

[…] some of the sickness you get it spiritually but it manifest out in a physical form so sometimes with the local one you try to get rid of the spiritual one from your leg yea you treat to get rid of the spiritual one from the leg and then from the hospital let them deal with the physical symptoms.

He felt there was a need to try many different traditional healers until you found the one that was able to suit your condition. When taking herbal treatment, he was cautious not to overdose and create further problem for his liver. One treatment was holy water, which healers “write items of the Quran on in ink”. He perceived that this could be used to drink and wash the body but he first boiled the water for infection prevention. The traditional healers also offered social support and rang the patient to check how he was doing. He suggested many ‘real’ traditional healers do not ask for money as they see their work as service to the community. This is well described in figure 6 titled *‘Never lose hope’,* showing both biomedical and local treatments and elements that give them hope.

### Trust in biomedical treatment

Participants spoke about the trust they had in biomedical treatment, seeing it as specialised, effective, and that the oncology unit staff were experts, “*whom we had belief in*”. For several, they chose to attend after advice from family members and husbands, acknowledging the good reputation it had and being specialist care.

Trust in biomedical care came after seeing improvements in their condition. This was helped by the counselling provided by the staff, who explained the treatment schedule and side effects, thus helping patients to remain adherent and to relieve the psychological burden.

Many patients were thankful to the staff who supported and operated on them. They spoke about the staff being friendly and a source of social support, using words like ‘family’ and ‘home’.

[…] and the oncology nurses, they were wonderful. They were great. Sometimes I just enter there and I'm crying they will sit me down, they will tell me we've been here for so many years. We have seen people who are out of cancer and you are one of them. You are one strong person we believe you can make it. So, they were also very helpful. – Boresa

Several patients chose to illustrate the medical support they received in their creative task. Tuurosung showed how she saw treatment as offering her hope by drawing a new shooting plant, with the treatment raining down on her and enabling her to grow (figure 3). Similarly, Zooya and Alamisi depicted medical elements that helped them cope with their condition.

However, relative of a patient with liver cancer, Asibit, perceived the treatment as ineffective and hopeless. He spoke about being prewarned that there was no hope for the patient to survive but it would take a large toll on them:

So my son told the mother that the thing is cancer. And she cannot survive it. So before we go to the hospital, we are going to waste money for nothing. There is no two ways about it that she can survive.

### Delayed medical diagnosis

For some, their trust in biomedical treatment had been initially strained by multiple misdiagnosis leading to delayed diagnosis of cancer at community and district facilities. This was a source of worry for most patients interviewed. Nabia was given ineffective medicine and initially treated with antibiotics. This was a similar case for Beteyang whose symptoms were initially treated with antibiotics and oral rehydration solutions, but when these symptoms repeatedly re-emerged, it was a source of worry. Tuurosung was often doing infectious disease tests, unsure of the cause of their symptoms, so the diagnosis and treatment brought relief. Napaga spoke of misdiagnosis causing delays of up to 15 years. Not knowing the cause of her symptoms was a major source of worry and debilitating. When she was initially found out about her suspected diagnosis at a private facility, she spoke about the distress she felt after poor counselling she received. *“The sickness! When it started it’s getting to fifteen years now, it first showed up through my genitals and I went to the [name] hospital severally ….”* - Napaga

Wumpagli spoke about how she was treated by a facility that did not conduct tests for cancer. The poor treatment was a source of frustration.

### Social support from family and friends

Several participants spoke about the social support offered by their husbands, wives and families. This came in many forms, talking, encouragement, pushing them to seek treatment, either biomedical or traditional, and through prayers. Some relied on their family as emotional support as were not able to talk to other people about their condition. Beteyang drew hope from their families support through encouragement and prayers. he spoke about how dark she felt and the bleakness (black colour) of diagnosis, by using black fabric. He illustrated this using multiple different shapes of different coloured and patterned fabric around the centre, to show the different psycho-social support that different members of their community had provided after the diagnosis (figure 4).

Boresa used her creative task to talk about the love and support patients need. She used a heart of pink beads to symbolise the need to have love and support (figure 2).

“I have a husband or a spouse who loves me and he tells me that I should fight. And that is why for me I have been able to fight up to this stage. The love; so every breast cancer patients needs love one way or the other.”

### Financial dismay

For some there was frustration that the medicines were anticipated to be covered by the NHIS, but the medicines were not available, so they had to pay for them, describing the NHIS as only a ‘name’. Participants also highlighted the lack of social security and support from non-governmental organisations, calling on the government give greater financial support.

You can’t come for the chemo on credit, you can’t run the tests on credit and there’s no support anywhere. Ideally we are supposed to follow the treatment scheduled, since I took the medicine I am fine, when I take chemo and return home it will weaken me but I can’t lay down, I have to force myself and go out to look for money … I don’t want to make a mistake and not follow the treatment schedule and all the money I spent go to waste. – Maakufaba.

The financial concerns included due to loosing work, the costs of tests, medical drugs, surgery-related costs and disposables patients required. For some this led to them delaying their treatment schedule. Bakpama accepted an interim treatment as he could not afford the consultant’s recommendations. Beteyang, on repeatedly being asked to buy medicines, described the medical professionals as ‘wicked’. The financial worry extended to the costs of tests, which also led to medical delays. Additionally, there was the cost of surgical equipment and disposable bags patients required. This led one patient to restrict their eating in a hope to reduce the financial impact she had one their family.

Maakufaba emphasised the need to save and the shame that can come from financial impairment from cancer using the creative task (figure 7).

The financial concern came from worry about where they would source the funds and having to borrow money from others. Some lent on support from family, but this was not always available. Participants borrowed money from local women’s group, local business owners and banks. However, this relied on their trust and being able to pay them back. Napaga was not able to pay the local women’s organisation back, which led to delays getting chemotherapy. Families sold their livestock to afford the treatment, but in some cases, the money raised was still not enough.

We paid huge sum of money. It wasn’t small amount, my husband sold four cattle he uses for ploughing during the farming season to pay for it. We don’t have anything again. We sold all our animals—goats and sheep’s. – Napaga

The financial burden was extended to relatives. For instance, Asibit talked about the costs of treatment, travel, blood tests and scans for the patient as a source of worry to his family. Recollecting, he felt dismayed by the ineffectiveness of the treatment and stressed the financial burden it had on them. *“Because you can't imagine the way we suffered with this lady. And finally, she just pass on like that. You can just imagine. And you look at the money that you have spent.”*

Similarly, Salifu spoke about his family borrowing money. He said he did not know how he would have enough money to survive in the future or continue to tertiary education.

Overall, the sentiment of all patients is well captured by the words of Alamisi *“[…]as for the cancer treatment if you don’t have money you will die […]”*

### Travel

Needing to travel for treatment imparted a greater financial burden. Alamisi and Maakufaba asked their husband and children to stop supporting them at clinics due to the travel cost. For Beteyang, coming from a rural setting far from the hospital, this led to challenges navigating the hospital and their systems.

Alamisi spoke of additional safety risks when travelling back from treatment late. She often mentioned the impact travel costs had on her. For example, when she was not eligible for chemotherapy one day, she worried how she would afford to return. She did not have anywhere to stay in the treatment city, so was frustrated when there were delays in her being seen to and commencing the treatment cycle. She indicated others in this situation sleep in the corridor. Salifu spoke about sleeping on the floor in the emergency room whilst he travelled to stay with their father. Alongside the mental burden and isolation, this gave him physical health concerns, contracting body rashes, and only being able to wash when a local seller offered them help.

### Non-disclosure of cancer

Many participants felt the need to be secretive about their condition. They hid this from customers and other community members. Some were open about their condition but spoke of other patients being secretive.

In some cases, this was linked to the cancer being seen as spiritual or caused by a curse. Asibit spoke about how he did not disclose the patient’s diagnosis to their community as he felt this could leave him susceptible to accusations. Family members of Nabia were surprised, as he was not seen as a problem maker. Zooya attributed the disease to being warned something evil would happen. *He said “they’ve already warned me that I shouldn’t come something evil will happen to me.”* – Zooya

There was also a notion that people would think it is contagious, according to Wumpagli, and further echoed by Nabia as *“in the wind”*.

The community perceptions were also linked to notions that cancer was seen as fatal. This was both by participants and their community members. Boresa remarked: *“[…] they scream, then look like you are a ghost who just died and came back to life.”* This impacted how their perceptions of identity and linked to concerns the cancer may return. Napaga revealed that members of her local community had announced her funeral whilst she is still alive.

Patients also experienced stigma due to their condition. Alamisi’s husband for instance, suggested she did not disclose her condition as would face insults and stigma due to having one breast. Bakpama spoke about how people stayed away from him due to the growth on their face and it led him to stop his farming work.

Negative perceptions of cancer patients also came from the burden that patients can impart on their family. *“I learnt most women once the person is diagnosed then her husband will pack her things before she gets home before she returns from the hospital. The husband will not want to take the burden”* – Boresa

### Cancer awareness

For some participants, they initially delayed seeking medical care as were not aware of their cancer symptoms. This was particularly the case for cancers less well known in the community. Boresa sought medical intervention after noticing her breast symptoms. However, she spoke about the need for her to be open about her condition to raise greater awareness. Maakufaba became aware of her symptoms after watching a TV show raising breast cancer awareness. Alamisi was sensitised to breast cancer after her mother’s death. However, patients with lymphoma, gluteal mass and head and neck cancer delayed seeking help as they were not aware that their symptoms could be linked to cancer.

## Discussion

This study demonstrates patients’ perspectives on cancer in northern Ghana and pinpoints elements that support and impinge staying in treatment. These elements will be critical for equitable policy intervention. The findings are building on existing understanding of factors associated with treatment drop-out in Ghana.^4^

The narrative interviews were found to highlight factors that were important to the patients in helping them continue to engage with treatment as well as barriers towards treatment engagement. The narrative interviews showed how the cancer treatment process intersected with their life experiences. This was built on with the creative exercise, which added more depth to their stories.

The creative exercise framed their experiences within what the patients and their families find important. It shed light on how the participant had processed and interpreted their experience, emphasising the unique message they wanted to share.

This study found a high psychosocial burden of cancer, which highlights the need for a holistic approach and that cancer needs to be viewed as a mental and physical illness. This aligns with cancer-specific studies, such as in prostate cancer, which showed the emotional impact on patients’ masculine identity.^31^ Whereas another study found high levels of comorbid anxiety and depression in patients with breast cancer.^32 33^

Patients will benefit from support to process their condition and to adapt to experience fulfilment in their lives. The findings align with others that demonstrate the importance of the psychosocial support offered by healthcare professionals.^33^ They underscore the need to embed holistic palliative services within oncology care in northern Ghana. At the time of this study, there was a small specialised palliative team (one nurse) serving the hospital. Expanding this service could ensure all patients have sufficient psychosocial support. Recent studies have demonstrated unmet needs in palliative care in southern Ghana and made calls for Ghana to integrate palliative care into primary health services.^34^ However, barriers exist at the individual and family, healthcare providers, institutional and policy level, requiring action.^35^

This finding also demonstrates the importance of community wellness and fulfilment through community contribution. Other research has suggested the strength of community ties in cancer.^36^ Mental health research in Ghana has suggested that patients may prioritise elements of social, spiritual and communal well-being.^37^ When this is not available through biomedical systems they may turn to traditional medicines.^37 38^

Indigenous models of wellness, incorporating precolonised trains of thought often include community well-being. The aboriginal definition, for example, considers health “*means not just the physical well-being of an individual but refers to the social, emotional and cultural well-being of the whole Community in which each individual is able to achieve their full potential as a human being thereby bringing about the total well-being of their community. It is a whole of life view and includes the cyclical concept of life-death-life.”*^39^ This indicates the importance of considering different models to interpret wellness and health in different cultures. Palliative care structures need to ensure they are appropriate to the local patients’ needs and reflect their cultural values.

Traditional understandings of wellness and high social cohesion could also be levered to offer holistic, community-based care. In other low-resource settings, a strong tradition of community has been harnessed to set up a community-led palliative care network.^40^

The prominence of meaning-making mechanisms suggests overlap with psychological models such as of Viktor Frankl, who used his traumatic experiences during the holocaust to understand how people find meaning in life. In ‘ On the meaning of life’^41^ he speaks about three sources of meaning—from a sense of duty or actions, including from a creator/God, through love, and in facing limitations and finitude. This aligns with how patients in this study found meaning to be resilient in the face of their condition.

Finding meaning through spirituality and religion was key for participants in this study. Spirituality, as a way of making meaning^41^ is often considered in models of palliative care. Extensive psychological research has identified positive impact of spirituality.^42^ Research in cervical cancer in Ghana has highlighted the spiritual needs of patients that are currently unmet.^43^

The results suggest training on cancer symptoms is needed at lower cadre facilities. This could limit psychosocial distress and support timely diagnosis. The findings highlight that financial challenges impart a high mental burden on patients and lead to stigma. Stigma has been linked to financial burden elsewhere, including in an African setting for cancer.^44^

Moreover, the financial coping strategies used, for example, loans and selling livestock may have implications on younger generations, breaking down household income sources. This could lead to poverty spirals and further instil stigma.^45^ Discussion with local oncology staff highlights that although there are charities able to support curative patients, they are unaware of organisations supporting palliative patients psychosocially or palliatively. This suggests palliative patients are underserved. Other studies have found financial challenges in patients accessing palliative care and called for its adoption in the NHIS.^34 35^

The results also suggest more consideration is required for patients travelling long distances to avoid multiple long journeys, leading to impoverishment. For example, scheduled appointment times, satellite clinics or remote testing could be helpful to ensure patients are able to minimise travel costs and maximise adherence to treatment.

Alongside financial burden, stigma has also been shown to have hallmarks of secrecy, self-causation and fatalism.^46^ Here, it was found that patients were secretive about their condition and there were beliefs in their communities that it was caused by a curse. This could cause stigma around the treatment. This has been found in other studies on cancer in Ghana, where it leads to mental distress.^35 47^ Stigma has been shown to lead to adverse treatment-seeking behaviours in other conditions and health inequalities,^48 49^ including for cancer in Africa.^44^ Fatalism has been linked to cancer stigma.^44^ Fatalism was found to be a belief about cancer here and has a long-standing tie to cancer, first articulated by Powe and Finne.^50^ This is supported by other recent research that has suggested fatalism and distrust lead to cancer treatment refusal in Ghana.^51^ They suggest fatalism is linked to poor communications and a breakdown in negotiating the patient pathway.^51^ This is in accordance with our findings, as opinions on and the support of health professionals influenced treatment behaviour. Moreover, the intersection between fatalism and feelings of futility in negotiating the pathway resonates with the concept of candidacy, used to better articulate the phenomena than ‘access’, by meaning along the full continuum of care.^4 52^

The study found the lasting impact of cancer on mental health in survivors, perceptions of cancer being fatal impacted how they perceived themselves and how they were perceived in the community. The term survivor can be applied in diverse ways, requiring clarity.^53^ Here, we considered survivor based on self-identification.

### Reflections on the research approach

Given the approach taken was novel in the setting, it was deemed important to critically reflect on this. Patients were recruited through nursing staff whom they had already built rapport with. This led to a high number of patients and relatives being comfortable to speak with the researcher. After the interviews, patients were followed up for feedback and several gave positive feedback that they were grateful their opinion had been heard. Acknowledging the psychosocial benefit, this has led to nurses seeking to follow up with patients regularly after treatment. Working directly with clinical professions at the oncology centre was helpful to gain the trust of the patients. Moreover, it enabled ongoing and responsive dialogue on the research findings. A gap in awareness of the oncology centre in rural regions had led staff to explore outreach activities in these areas.

Regarding the creative task, overall, the participants engaged well and feedback suggested that it made them process their experience in different ways, to reflect on their experiences and to understand their condition and coping mechanisms. We found the artwork was resonant, with a clear strong message, emotive and personalised. The visual elicitation using fabric crafts highlighted concepts and experiences that were important to patients and framed their story within their unique social perspectives, giving greater contextuality. The strength of the pieces goes beyond viewing patients as cases, but as within their unique lives this is more humanistic and allowed the researcher to relate to the patient. The images show how life trajectories intersect with cancer treatment and lead patients to process their condition and find meaning in different ways. This is hoped to allow policy-makers and members of the public to relate to their experiences and instigate changes to improve conditions. This has led to an exhibition of the artworks at the oncology centre being planned. This is hoped to provide impact of the research beyond knowledge generation. Showcasing the artwork can support greater understanding and motivate future patients to adhere. This aligns with the transformative paradigm, viewing the research as an agent for change^9^ and went beyond ‘do no harm’ ethics to impart positive impact.^54^ Extensive evidence suggests a positive impact of art-based therapy in palliative care.^55^

### Strengths and limitations

This is the first qualitative study covering a large range of cancers and in northern Ghana.

The study has contributed to methodological advances. A new approach combining narrative interviews and graphic elicitation was pioneered. It highlights this approach is highly informative, giving rich perspectives and the participants’ frame of the treatment process. This aligns with findings on similar approaches in other settings.^21^ The method was found to lead to much more humanistic and resonant data. This is anticipated to be of greater utility to discuss with policy-makers.^56^

The analysis approach was first inductive, a decision made considering the lead authors positionality. One limitation is the requirement for translation in some instances. Language is entrenched in power hierarchies leading to English views of evidence dominating knowledge constitution.^57^ In attempts to decolonise the approach taken here, other types of evidence, using arts-based method were innovated.^58^ The approach also considered the positionality of language and how translation is influential within the research.^57 59^

Patients with cancer are often stigmatised, and the approach required sensitivity and to gain their trust to speak, so initial contact was made through a health worker they had already encountered. Although this introduces a risk of selection bias, it was felt critical to be sensitive to patients’ needs and avoid undue harm.

Using the RTA approach, the researchers’ subjectivity was seen as a resource, rather than an element to be neglected and minimised.^24^ The lead author was from outside of Ghana, but spent and extended period living in Ghana and volunteering at the oncology centre, and had multiple ongoing discussions about the research with local health professionals and researchers. This is thought to have enabled them to gain a better understanding of the cultural context, while also acknowledging their limitations as an outsider. It can be argued that an outsider perspective can pick up on nuances others may miss.

Additionally, a limitation was that we were not able to recruit a liver cancer patient, despite the high burden of disease identified.^22 23^ This indicates a missing voice that future research should look for ways to incorporate.

## Conclusions

The findings demonstrate a novel, effective approach to shed an in-depth understanding of cancer experiences in an African setting. It highlights factors deemed as important to patients and their family. In particular, it shows there is a need for policy-makers to take more holistic approaches to cancer patient well-being—considering their psychosocial, spiritual and occupational fulfilment to help them adhere to treatment. NHIS policy should prioritise the costs of diagnostic tests and treatment for better, more equitable, outcomes. The highly resonant nature of the visual findings suggests an effective way to communicate these needs with policy-makers.

## supplementary material

10.1136/bmjopen-2024-093303 online supplemental file 1

10.1136/bmjopen-2024-093303 online supplemental figure 1

## Data Availability

Data are available on reasonable request.
