# Supplementary material for: What are the lived experiences of patients with cancer and their families in northern Ghana? A qualitative study using narrative interview and creative task approach
Source: BMJ Open. 2025 Feb 26;15(2):e093303. doi: 10.1136/bmjopen-2024-093303 (PMC11865803; doi:10.1136/bmjopen-2024-093303)
Supplement: online supplemental file 1 [file bmjopen-15-2-s001.docx]

**Narrative interview with arts-based elicitation task (graphic elicitation technique)**

More details on the method is provided for interpreters at the end of this document.

**Informal interview guide**

**Introductions**

**Introduction to interviewer and their work**

Introduce researcher role, their positionality (who they are), why they are choosing to do this research

**Introduce research**

*I wanted to talk to you today about your experiences with cancer, this will involve a chat where you can say as little or as much as you like about your experiences and what is important to you around these experiences.*

*Then, if you are comfortable, we will take part in a simple collage activity to explore this more in a creative way.*

*You can take a break or stop at any time.*

*In total it will likely last around 1 hour.*

*We’ll also record the interview so we can remember everything we talk about.*

Check they are comfortable with this before starting

**Participant introduction**

Note: I first think it is important to make sure they have the opportunity to feel more than a cancer diagnosis. Also, setting the context can help frame and get a better understanding of their social position and potential influence

Example questions to start conversation:

*But firstly, before we talk more about that it would be great to hear about you, your name is?*

*Do you like to be called that, or do you have another name?*

*Where are you from, did you grow up there?*

*With your family, are you close? Are they from ….. also?*

*Did you go to school there? Work?*

*So now, you’re speaking….., but do you speak other languages too?*

*And now, where are you living?*

*And do you have any interests?*

*What about drawing, sewing, fabrics, fashion? Cooking? Sports?*

**Part 1 - Narrative**

**Explain the narrative approach to the participant**

Example:

*So, next I’d like to ask you a few questions about your cancer diagnosis, but more than being led by me, it’s an opportunity for you to share whatever you’d like to share. So stop me anytime, you’re the driver here not me, as it’s your experience so you are the expert.*

*There are no right or wrong answers, I just want to learn about your experience.*

*You don’t have to answer the questions if you prefer not to and we can stop the interview or pause at any time.*

***Prompts***

*So when did you first notice your cancer?*

*Where were you then?*

*What did you do? Did you talk with anyone?*

*Then what happened?*

*So, is that a district hospital / spiritual centre / …..?*

*Tell me more about that, did it help?
What after that?*

*Tell more more, did that help?*

*Can you explain more about that, your time there, what was involved?*

*What do you remember most about that?*

*Anything else?*

*Is there anything else you would like to say about these experiences?*

*Has it changed how you see yourself?*

*In what way?*

*Can you expand?*

*Do you think it affects how other people see you*?

**Part 2 - graphic elicitation**

**Explain the arts task to the participant**

For example:

*For the next part, the idea is to create something. Often only using questions can be restrictive, especially because they all end up in English, which doesn’t seem fair. There are lots of other ways to convey messages, pictures and art can be really powerful too. Some people also find it helpful to make sense of things. I’ve noticed in Ghana the beautiful fabric work is really powerful to express things.*

*I’ve brought some pens, fabrics, thread and these hoops. [show embroidery hoop but no demo version as this can be limiting]*

*You can put the plain fabric inside this hoop and use it to create something - it can be simple but still tell your story and express some part of how you feel.*

*If you’d like, we would like you to create something that represents any aspect of your experience with cancer or anything related to this that you would like to share with others (the staff, the people who make decisions, family, community…). It can be something we talked about or something else.*

*You can use this hoop as a base and then add things - by sticking or sewing or drawing on it (or a mixture of any of these - there are no rules here). Add whatever you like to create your piece.*

*It is not about the way it looks but about expressing yourself. There is no wrong or right way to make the art, it is just whatever is helpful for you to express yourself. You even find new ways that we didn’t know and teach us.*

*Whether it looks good in the end does not matter. It’s also meant to be something very relaxed and we hope you can enjoy taking part.*

*When you have finished making your image give it a title. Then we will have some time to talk together about what you’ve made and how you found this experience.*

*You can say as little or as much as you want about your image.*

*So if you’d like to try this, it’s completely your choice. ‘You can also stop at any time.*

*It’s really no pressure, so long as you are happy and comfortable.*

**Prompts during the creative process (if required/time allows):**

*How is your artwork going?*

*There are lots of different fabrics here, you can include them either for visual effect or they can be symbolic too. And cut them up, rip them, whatever, it’s for you to use.*

*These pens and paints, can be for drawing or writing - you can practise here too*

*There is also thread, lots of different colours - in case you would like to sew - this can be a pattern or writing.*

*Whatever you feel most comfortable using.*

**Part 3 - feedback on the elicitation task**

In this section use the questions to ask about their artwork:

*This is looking great, is it finished?*

*What title would you give this?*

*Can you talk to me about this? You can say as little or as much as you like.*

*So is that important for you?*

*That’s interesting, was there any reason you picked these colours, fabric composition?*

*What is this you’ve drawn?*

*Can you explain a little more about that?*

*How did that come about?*

*How was that for you?*

*What does…. Here mean, can you explain?*

*How would you caption this?*

*Is there any take away message to share with others from this?*

*Would you prefer to keep it private or share it?*

*Who would you like this to be shared with, if possible?*

*Is there anyone you wouldn’t want to see this?*

*Would you like to keep it, or would you like me to keep it so we can include it in an exhibition we will present to cancer decision makers?*

**Closing the interview**

Thank the participant and ask them for any further feedback or if there is anything they would like to share.

*Thank you so much for taking part today, I’ve really enjoyed talking to you and learned a lot.*

*I’m very grateful for your help as I appreciate it can be very challenging talking about cancer.*

*How did you find it?*

*That’s good to know, we will take that on board, is there anything else?*

*How about the art task? This is quite a new approach so we are keen to get feedback.*

*Is there anything else you’d like to raise that we’ve not yet talked about?*

*So the interview and task are going to be included in my research to help understand and improve cancer experiences here.*

*Thank you again, so much for your time.*

**After the interview**

Arrange a follow up call to be conducted by a member of staff to ensure they arrived home safely and they comfortable after the discussion. Additionally ask for any further feedback.

**Introduction to the methods for interpreters**

Narrative interviews have been found to provide rich data that privileges aspects important to the participant. Patient’s narratives can reflect how they identify with and make sense of their condition and expand understanding outside of a clinical focus to psychosocial, spiritual and community factors that also influence their beliefs and interactions with healthcare (Bissel, 2006). Additionally, narrative approaches can be more sensitive as it allows them to explore distressing topics as they choose, which has been found to be insightful studying cancer (Overcash 2003).

Narrative approaches can vary in terms of their levels of freedom and direction by the interviewer. In healthcare, the narrative interview typically sets a flexible frame around the health condition/phenomena in focus (Ryan 2007, Anderson and Susan Kirkpatrick, 2016).

It is important that any interview approach is sensitive to the participants' needs, as cancer can bring with it many strong and hard to articulate emotions. Moreover, the researcher- participant power dynamic can be daunting. Approaches such as art therapy can help patients make sense of this and express inner experiences that they may struggle to put into words in a more relaxed environment (Wiley handbook of art therapy, Gussak and Rosal, 2016).

Recognising the limitations of language to articulate experiences, the power dynamics between the researcher and participant and sensitivity needed with cancer, cancer narrative interviews have been built upon to include creative methods such as art elicitation tasks (graphic elicitation technique) (Mooney 2014).

Graphic elicitation is an established technique,(Bagnoli 2009, Umoquit, 2011, Orr 2020) this is even more suitable for my research with patients in Ghana as it is known to be helpful in cross-cultural settings and when language is limiting (Kara 2015). This can involve a variety of different visual art forms. Collage is an art form that can be empowering as there is no artistic limitation like drawing (Malhotra et al. 2021).

A methodology to combine collage graphic elicitation with interview approaches has been well defined in health research by (Malhotra et al. 2021).  They trialed the approach in patients experiencing gulf war syndrome and found the methods help to bring focus to salient points of their condition and reveal unarticulated experiences such as mental states, hopes and coping skills. It provided enriched explication through facilitating deeper reflection on mood and emotions. My approach draws on the approach they took. However, it is important that creative methods are context-informed (Kara, 2015,Gussak and Rosal 2016). Given the rich culture of fabric work and experience participants in Ghana may have with this, incorporating fabrics may offer an approach participants feel comfortable with. In the art therapy discipline, use of fabrics has been found to help personal expression, improve communication and meditative skills (Gussak and Rosal 2016). The approach taken here will involve mixed-media fabric collage with needle work.

Malhotra et al. 2021’s approach was overseen by art therapists and researchers were trained to ensure participant safety given the strong emotions that can be evoked from distressing topics.

The approach here has considered sensitivity to patient needs through several approaches:

- The draft guide to be reviewed by a practising art therapist in the UK
- The draft guide to be reviewed by a oncology nurse working at TTH
- The researcher has experience running art workshops with participants (made vulnerable) in the UK
- The researcher will first spend several weeks volunteering in an oncology setting in Tamale to sensitise themselves to the patients needs
