# Supplementary material for: What are the lived experiences of patients with cancer and their families in northern Ghana? A qualitative study using narrative interview and creative task approach
Source: BMJ Open. 2025 Feb 26;15(2):e093303. doi: 10.1136/bmjopen-2024-093303 (PMC11865803; doi:10.1136/bmjopen-2024-093303)

**Supplementary figures**

Supplementary Figure 1: Untitled

Salifu, a son of a patient who had liver cancer uses the creative task to depict changing family dynamics as father become ill and children take own financial and caring roles.


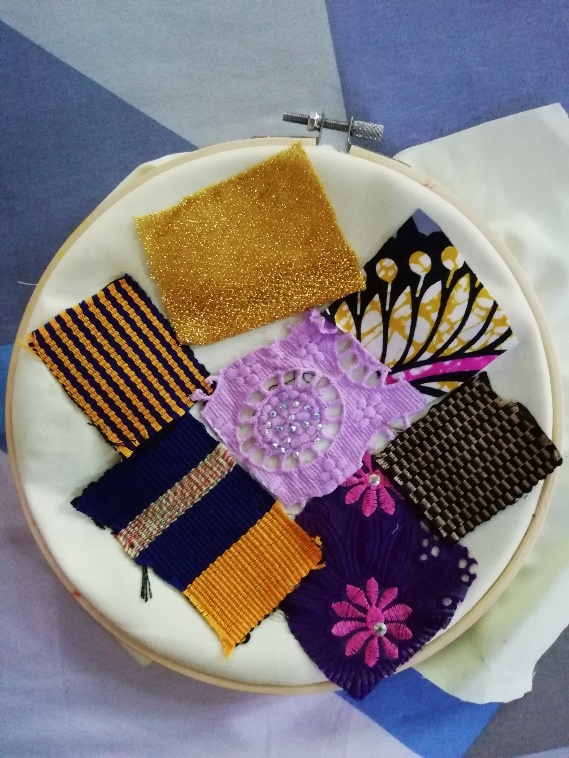


Supplementary Figure 2: Untitled

Naazo chose traditional smock fabric which they identified with, to articulate their situation. *‘This is my God, this is the sickness, this is peace, this is my family, and I am this.’*


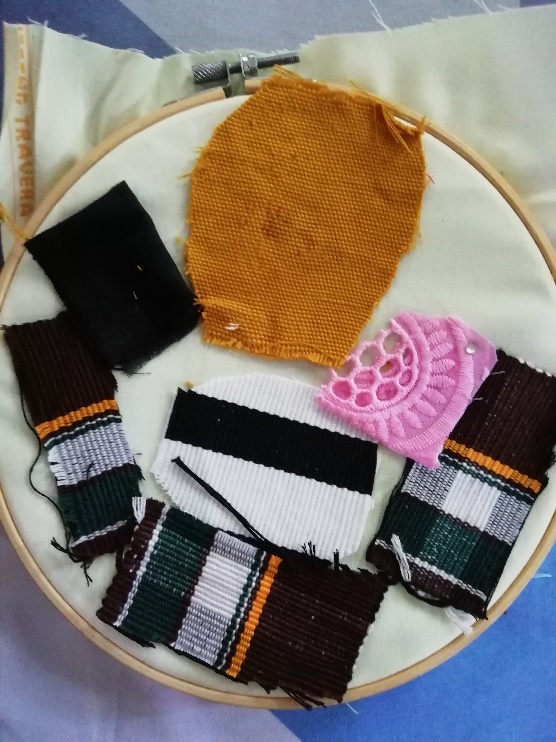


Supplementary Figure 3: *With God all things are possible. What God cannot do does not exist*.

Relative of cancer patient Tipagya, shares a message of faith in God which was helping her cope with the situation


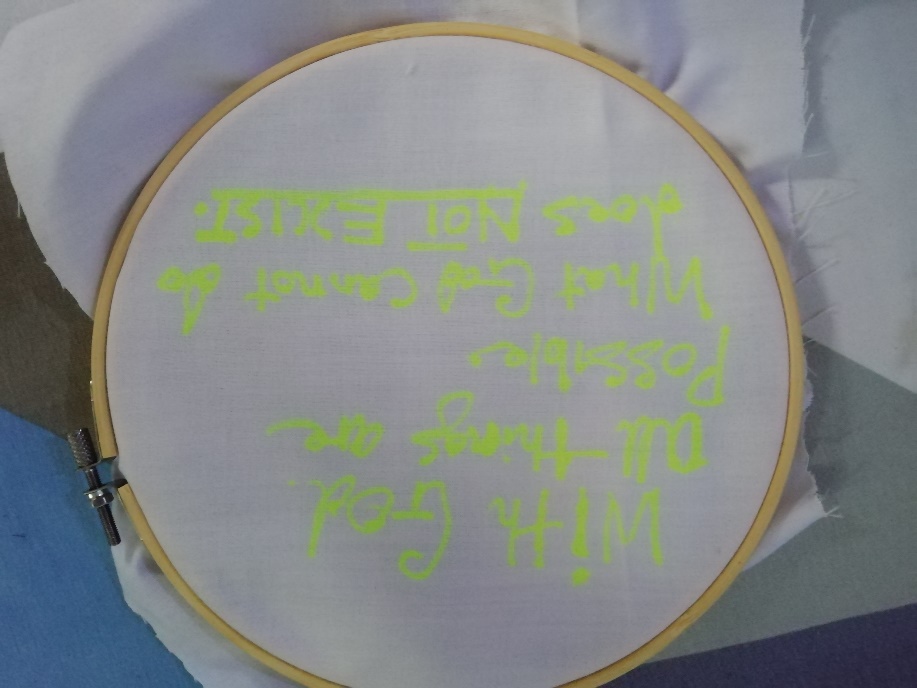

Supplement: online supplemental figure 1 [file bmjopen-15-2-s002.docx]
