# Supplementary material for: What are the lived experiences of patients with cancer and their families in northern Ghana? A qualitative study using narrative interview and creative task approach
Source: BMJ Open. 2025 Feb 26;15(2):e093303. doi: 10.1136/bmjopen-2024-093303 (PMC11865803; doi:10.1136/bmjopen-2024-093303)
Supplement: Supplementary file 3 [file bmjopen-2024-093303.reviewer_comments.pdf]

PEER REVIEW HISTORY

BMJ Open publishes all reviews undertaken for accepted manuscripts. Reviewers are asked to complete a checklist review form and are provided with free text boxes to elaborate on their assessment. These free text comments are reproduced below.

ARTICLE DETAILS

Title (Provisional)

What are the lived experiences of cancer patients and their families in northern Ghana? A qualitative narrative interview and creative task approach

Authors

Tuck, Chloe Zabrina; Akparibo, Robert; Gray, Laura A; Suraj, Hamza; Iddrisu, Abdul-Rashid Timtoni; Abane, Tampuri Rahman; Deedat, Alhassan Ahmed; Aryeetey, Richmond Nii Okai; Azure, Amos; Cooper, Richard; Abubakari, Braimah Baba

---

VERSION 1 - REVIEW

**Reviewer**	**1**
**Name**	**Okyere, Joshua**
**Affiliation**	**University of Cape Coast, Department of Population and Health**
**Date**	**18-Nov-2024**
**COI**	**None**

Thank you for the opportunity to review this important manuscript. While this manuscript is well-written, there are some points to be improved.

Introduction

1. The first paragraph will benefit from significant improvements. The authors stated: "Cancer poses a huge burden on health and wellbeing globally, which is increasing in many low and middle income settings, including Ghana, where it typically receives less attention, especially policy prioritisation". However, how serious is the issue? Providing some statistics from Ghana will better situate the background provided.

2. The authors stated that cancer has received less attention especially in policy prioritisation. I would urge the authors to be careful with the choice of words. Generically stating that cancer has received less attention in Ghana is erroneous. Cancers like breast, cervical and childhood cancers have received significant attention policy attention in Ghana. So, please review literature appropriately and provide enough policy context.

3. Again, the first paragraph can be divided into two paragraphs. The first will focus on the gravity of cancers in Ghana. The second paragraph will then elucidate the factors that influence healthcare services for persons living with cancer.

4. "As studies were centred in southern Ghana, less was known about Northern Ghana, where socioeconomic status and culture may influence treatment behaviours and beliefs". The authors stated this with not evidence. Please provide citations to support your argument.

5. "In unpublished cross-sectional analysis from another stage of this research we have identified treatment incompletion as a major concern in Northern Ghana". These statements seem factual so please provide evidence.

6. "This qualitative study was conducted as part of a larger mixed methods study to explore factors that influence cancer treatment uptake, and identify strategies to increase treatment uptake in Ghana". This statement should be in the methods section.

7. "This is for all types of cancers, but there was a large amount of missing data for some cancers". What study are the authors referring to? If the study is based on the findings of a particular study, then the study should be cited appropriately.

8. Reading the introduction, I get the understanding that the authors are interested in understanding the reasons for treatment incompletion for persons living with cancer. If that is correct, then the authors should situate the entire introduction around that.

Methods

9. What do the authors mean by this statement: "The research methodology was situated within the social justice paradigm and sought to uncover the contextual richness of social experiences"? Note that your methodology is also literature for other researchers. Kindly expand the statement.

10. I am wondering. The authors state that their study is based on a larger mixed method study. Did the quantitative component investigate treatment completion rate and its associated factors? If yes, why did the authors decide to present only the qualitative findings? My question is based on evidence from the manuscript that the authors consistently make reference to the larger study/cross-sectional quantitative survey.

11. How was the sample size determined? What was the stopping criterion?

12. What analytical framework was followed in the reflexive thematic analysis?

13. Who among the authors performed the analysis and how were coding differences resolved?

14. "The interview process, and approximate timings, were explained at the outset." Be cautious about the use of punctuation. The two commas in this sentence are not needed. It makes reading difficult.

15. This statement is not clear: "Fifteen adult (>18 years) patients or their relatives who had been diagnosed and/or treated for cancer at a teaching hospital in Northern Ghana within the last 2 years were purposively sampled". Do you mean you recruited patients or relatives of patients, or relatives who have been diagnosed with cancer?

16. What was the eligibility criteria for selecting relatives?

17. The authors stated that 8 patients alone and 5 with relatives present. But that gives us 13. So, how come it is reported that 15 interviews were conducted?

18. "Participants were invited and sensitised to the research by a local nurse who they already had contact with". This methodology introduces selection biases. How did you ensure that the nurses' involvement in the study did not unduly influence participants' decision?

19. In what language were the interviews conducted? Did the authors translate the interview guide? Please attach your interview guide as a supplementary file.

20. "Interviews were transcribed verbatim by the research assistants". Who were the research assistants? How many were they? What training was given to the research assistants?

21. Include a section on rigor and trustworthiness.

22. Include a section on reflexivity.

The creative analysis is a significant contribution to literature. Kudos to the authors for that.

**Reviewer**	**2**
**Name**	**Salisu, Waliu Jawula**
**Affiliation**	**Cambridge University Hospitals NHS Foundation Trust**
**Date**	**31-Dec-2024**
**COI**	**None**

Thank you for the opportunity to review such an interesting manuscript.

The research topic is highly relevant and uses a novel approach which was implemented impressively. The manuscript is well-written and thoughtfully articulated.

However, I would like to suggest a few areas where the authors might consider revising for clarity and specificity.

Methods

The statement "Using both collage and interview methods together can to bring focus..." appears unclear.

The statement "Secondly, the lead author spent several weeks volunteering in an oncology setting in Northern Ghana to familiarise her to the patients' needs. The lead author and worked with...." could benefit from a revision to correct grammatical errors and if possible clarify the lead author's specific activities and their relevance to the study.

Data Analysis:

The phrase "Themes were identified from the transcripts inductively, rather than being guided by existing frameworks that may overlook data if they are take..." could be reworded for clarity.

Findings:

The sentence "Of this five, one patient allowed their relative to conduct the interview and creative task on their behalf..." seems unclear. Did the patient permit their relative to conduct the interview on their behalf, or was the relative simply participating? It would also help to clarify whether the authors were the ones conducting that particular interview or the participants.

Discussion:

This section is well-written and supported by appropriate references.

However, could I suggest that the statement "Currently they have a small specialised palliative team (one nurse) serving the hospital." be reworded to something like ..."at the time of this study" This is just to clarify to readers and also enhance the credibility of the study as the team would have currently outgrown the "single nurse team" and anyone reading would appreciate this was only at the time of the study.

References to Fatalism:

The statement "Fatalism has been linked to cancer stigma (38). Fatalism was found to be a belief about cancer here and has as a longstanding tie to cancer, first articulated by Powe and Finne(44)." I think this statement could be further supported by citing a more relevant reference such as

(https://pmc.ncbi.nlm.nih.gov/articles/PMC9040785/#sec17) which is directly related to the context and findings of this study. I think this could strengthen the connection to the current research context and add depth to the findings and discussion.

VERSION 1 - AUTHOR RESPONSE

Reviewer 1		
Thank you for the opportunity to review this important manuscript. While this manuscript is well-written, there are some points to be improved.	Thank you for your encouraging feedback and helpful points.	

Introduction<br>1. The first paragraph will benefit from significant improvements. The authors stated: "Cancer poses a huge burden on health and wellbeing globally, which is increasing in many low and middle income settings, including Ghana, where it typically receives less attention, especially policy prioritisation". However, how serious is the issue? Providing some statistics from Ghana will better situate the background provided.	Thank you, we have added additional references to clarify this.<br><br>*According to Global Health Observatory estimates, there were 15,802 cancer-related deaths in 2020 (based on figures released in 2022), with liver, breast, cervical, and prostate cancers contributing the greatest burdens (1,2). By 2024, this number had risen to 17,944, with liver cancer remaining the leading cause of death (3). While the prominence of breast and cervical cancers is increasing, many other cancers receive relatively less attention, particularly in terms of policy prioritization, as highlighted and discussed in our previous literature reviews (2,4).*	3
2. The authors stated that cancer has received less attention especially in policy prioritisation. I would urge the authors to be careful with the choice of words. Generically stating that cancer has received less attention in Ghana is erroneous. Cancers like breast, cervical and childhood cancers have received significant attention policy attention in Ghana. So, please review literature appropriately and provide enough policy context.	Thank you for this. We agree the policy landscape is complex and further clarification would be helpful on this. Our previously published literature review highlighting the lack of attention cancers other than breast and cervical generally get in adults. Whilst we agree that there is a growing interest, and recently some political will has been demonstrated to address some cancers in Ghana these have not yet been translated into actions to fully tackle the problem.<br>We've added further clarification regarding the attention received and references to our literature reviews (Tuck et al., 2022 BMJ Open and Tuck et al., 2023 Int J Equity Health) in the manuscript.<br><br>*While the prominence of breast and cervical cancers is increasing, many other cancers receive relatively less attention, particularly in terms of policy prioritization, as highlighted and*	3

	discussed in our previous literature reviews (2,4).	
3. Again, the first paragraph can be divided into two paragraphs. The first will focus on the gravity of cancers in Ghana. The second paragraph will then elucidate the factors that influence healthcare services for persons living with cancer.	Noted and amended	3
4. "As studies were centred in southern Ghana, less was known about Northern Ghana, where socioeconomic status and culture may influence treatment behaviours and beliefs". The authors stated this with not evidence. Please provide citations to support your argument.	Apologies if this was not clearly articulated, this relates to the reference noted above (Tuck et al., BMJ Open 2022) – we have articulated this in the text.<br><br>*Findings from our earlier review of the literature found that although there was a substantial amount of literature on breast and cervical cancer, there was a dearth of information on what influenced treatment completion for other cancers(4).*	3
5. "In unpublished cross-sectional analysis from another stage of this research we have identified treatment incompletion as a major concern in Northern Ghana". These statements seem factual so please provide evidence.	Thank you for highlighting, this has now been published so we have referenced.<br>*In a published cross-sectional analysis from an earlier stage of this research, we identified treatment incompletion as a significant concern in northern Ghana (8).*	3
6. "This qualitative study was conducted as part of a larger mixed methods study to explore factors that influence cancer treatment uptake, and identify strategies to increase treatment uptake in Ghana". This statement should be in the methods section.	Thank you, we have moved this to methods as suggested.	4
7. "This is for all types of cancers, but there was a large amount of missing data for some cancers". What study are the authors referring to? If the study is based on the findings of a particular study, then the study should be cited appropriately.	Thank you, this is now cited.<br><br>*The cross sectional study found missing data for all types of cancers, with particularly large gaps for certain cancers other than breast and cervical cancer (8).*	4
8. Reading the introduction, I get the understanding that the authors are interested in understanding the reasons for treatment incompletion for persons living with cancer. If that is correct, then the authors should situate the entire introduction around that.	Thank you, we have clarified this by framing the introduction with more text on this explicitly.<br><br>*Therefore, in the current study we aimed to explore the reasons for non-completion in this setting*	4

	using a qualitative approach. Thus, the research question addressed was: What are the individual stories of adults with cancers and how do they interact with services in Northern Ghana?	
Methods<br>9. What do the authors mean by this statement: "The research methodology was situated within the social justice paradigm and sought to uncover the contextual richness of social experiences"? Note that your methodology is also literature for other researchers. Kindly expand the statement.	We have added some further clarification around this.<br><br>*This paradigms accommodates various forms of evidence, recognising the social, cultural and historic contexts influences the priority given to each (9,10). Different types of evidence play distinct roles in driving social and political change. This study applied qualitative methods to uncover and provide a rich contextual understanding of the social experiences of living with cancer.*	4
10. I am wondering. The authors state that their study is based on a larger mixed method study. Did the quantitative component investigate treatment completion rate and its associated factors? If yes, why did the authors decide to present only the qualitative findings? My question is based on evidence from the manuscript that the authors consistently make reference to the larger study/cross-sectional quantitative survey.	Thank you for highlighting.<br>We have added a reference to the quantitative part which is now published.<br>As both studies were substantial and took different approaches and questions using novel methods that require explanation it was felt most appropriate to publish separately.	
11. How was the sample size determined? What was the stopping criterion?	This has been clarified.<br>*Sampling was stopped after 15 participants as theoretical saturation was reached.*	6
12. What analytical framework was followed in the reflexive thematic analysis?	We have added further clarification that we followed the six stages of RTA:<br>*This involves six stages: first familiarisation with the data, followed by initial coding. The codes are then group together to form initial themes, which are reviewed and reassessed. The themes are refined, and names are assigned. Finally, each theme is articulated in the write up. This analysis conducted manually to allow greater immersion in the*	7

	text, using multiple colour highlights and mapping.	
13. Who among the authors performed the analysis and how were coding differences resolved?	Thank you for this helpful suggestion. We have added greater clarity on this.<br>*Initial codes were generated by CT and reviewed either by RA and RC. Any discordance in codes were resolved through discussion. CT also developed the initial themes which were reviewed and discussed with RA, RC and LG. It was not feasible to share the transcripts and findings with participants due to low literacy rates. However a graphic booklet is being developed alongside this research, which incorporates participants feedback.*	8
14. "The interview process, and approximate timings, were explained at the outset." Be cautious about the use of punctuation. The two commas in this sentence are not needed. It makes reading difficult.	Thank you, noted and extra commas removed for clarity.	7
15. This statement is not clear: "Fifteen adult (>18 years) patients or their relatives who had been diagnosed and/or treated for cancer at a teaching hospital in Northern Ghana within the last 2 years were purposively sampled". Do you mean you recruited patients or relatives of patients, or relatives who have been diagnosed with cancer?	Thank you, we have rephrased this for clarity<br>*Fifteen adult (>18 years) patients who had been diagnosed and/or treated for cancer at a teaching hospital in the north of Ghana or the relatives of such cancer patients, treated within the last 2 years, were purposively sampled.*	5
16. What was the eligibility criteria for selecting relatives?	Thank you, we agree this is a helpful addition:<br>*This followed the same inclusion parameters as patients; however, instead of being a patient treated at the site within 2 years (over 18 years), they were individuals who had regularly accompanied a patient for treatment during that time frame.*	6
17. The authors stated that 8 patients alone and 5 with relatives present. But that gives us 13. So, how come it is reported that 15 interviews were conducted?	Thank you, although this is clarified in the table, we can see that this may be unclear. Thus, we have ensured it is clearer in the text as well to avoid confusion.<br><br>*Fifteen interviews were conducted with patients and or their relatives.*	10

	Eight interviews were with patients alone, five patients also had relatives present, while two were with relatives only. Of the five interviews with patients and relatives, most involved joint participation of the patient with their relative, except, one patient who allowed their relative to solely partake in the interview and creative task on their behalf. On one occasion a relative stepped in to partake in the creative task only. The patient pool is summarised in Table 1.	
18. "Participants were invited and sensitised to the research by a local nurse who they already had contact with". This methodology introduces selection biases. How did you ensure that the nurses' involvement in the study did not unduly influence participants' decision?	We agree there is a risk of bias here, but it was also felt necessary in order to carry out ethical and sensitive research. We have added a statement to discuss this point in the discussion.<br><br>*Participants were invited and sensitised to the research by a local nurse who they already had contact with, using the information in the participant information sheet.*<br>....<br>*Cancer patients are often stigmatised, and the approach required sensitivity and to gain their trust to speak, so initial contact was made through a health worker they had already encountered. Although this introduces a risk of selection bias, it was felt critical to be sensitive to patients' need and avoid undue harm.*	6 and 27
19. In what language were the interviews conducted? Did the authors translate the interview guide? Please attach your interview guide as a supplementary file.	Thank you for highlighting this was not more explicit, we have further articulated this.<br><br>*Data collection took place at the hospital's Oncology unit, from July to October 2023, using an interview guide (see supplementary information).*	6

	Interviews were conducted in the preferred language of the participant, and include: English, Dagbani, Kusaal or Twi. The lead author (CT) led with the interview in English, which were translated to other languages by a pre-selected interpreter (qualified male nurses with oncology experience, HS/AA). The interview guide was made flexible to accommodate participants reflections, questions, as well as it allowed the interviewer to clarify doubts. It included an introduction which explained the aims of the research and the lead researchers' reasons for pursuing the study.	
20. "Interviews were transcribed verbatim by the research assistants". Who were the research assistants? How many were they? What training was given to the research assistants?	We have provided additional information on this for your clarification.<br><br>*Interviews were transcribed verbatim by three transcribers (post graduate students experienced in transcribing, and briefed on the research and task) and validated by the lead author (CT). Where required, translation was conducted by the authorship team and discussed with to resolve discrepancies.*	7
21. Include a section on rigor and trustworthiness.	Thank you, this was touched on in the discussion, but we have added to the methods for clarity.<br>*Trustworthiness*<br>*This approach incorporate several features anticipated to create trustworthiness in qualitative research – credibility, transferability, dependability, and confirmability(29,30). Credibility relates to the research as an authentic representation of reality, which was achieved through peer debriefing, thematic saturation and triangulation of results. After each interview, the lead author held debriefed meetings with the interpreters to highlight strengths, limitations and where*	9

	improvements in the approach could be made and minimise bias (confirmability). Dependability was created through transcription, meetings notes, and an audit trail. Qualitative research seeks transferability, rather than external validity, which was sought through rich description and combining both speech and visual evidence to provide greater context.	
22. Include a section on reflexivity.	Thank you, again, this was in the discussion, but has been added to the methods so is clear to readers that it was considered from the outset.<br>*Positionality and reflexivity*<br>*A researchers' subjectivity is inherent in reflexive thematic analysis. Although the lead researcher (who collected and analysed the data) was from outside of northern Ghana, she lived and volunteered in the setting and worked closely with healthcare professionals to improve their understanding of the local context. During data collection CT (a white female researcher), who lead in the interviews acknowledged the complexities of what her identity may bring to the research, particularly when interviewing non-white participants. She was aware that her position is shaped by her cultural background, upbringing, and experiences, which may differ significantly from those of the participants, whose lived experiences are informed by different cultural, racial, and social contexts. It also important to recognize how these differences may influence the ways in which she engages with the participants, interpret their responses, and make sense of their experiences during analysis of the data. CT was sensitised to academic and literacy*	9

	writing on colonialism and positionality, to establish a stronger, critical acknowledgment of the colonial legacies that have shaped international research and reflect on how that may impact how they are perceived.<br>*Whilst CT acknowledges her subjective interpretations are positioned as an outsider, it was felt this also lent to broadening perspectives of the findings.*	
The creative analysis is a significant contribution to literature. Kudos to the authors for that.	Thank you for your positive feedback!	
Reviewer 2		
Thank you for the opportunity to review such an interesting manuscript.<br>The research topic is highly relevant and uses a novel approach which was implemented impressively. The manuscript is well-written and thoughtfully articulated.<br>However, I would like to suggest a few areas where the authors might consider revising for clarity and specificity.	Thank you for your helpful and encouraging feedback	
Methods<br>The statement "Using both collage and interview methods together can to bring focus..." appears unclear.	Thank you for spotting this, we have reworded.<br>*Using both collage and interview methods together can bring greater focus to salient points of a patient's condition*	5
The statement "Secondly, the lead author spent several weeks volunteering in an oncology setting in Northern Ghana to familiarise her to the patients' needs. The lead author and worked with...." could benefit from a revision to correct grammatical errors and if possible clarify the lead author's specific activities and their relevance to the study.	Thank you for spotting this typo. We have amended and added further clarity to this section.<br><br>*Secondly, although the lead author (CT - who led the interviews, and data analysis) was from outside of Ghana, they spent several months volunteering at the hospital's oncology centre in the northern region of Ghana where this research was carried out. This allowed CT to familiarise herself with the patients' needs.*	8
Data Analysis:<br>The phrase "Themes were identified from the transcripts inductively, rather than being guided by existing frameworks that may overlook data if they are take..." could be reworded for clarity.	Thank you, we have reworded for clarity:<br><br>*This decision was taken as frameworks may overlook some evidence if they are take*	7/8

	perspectives exclusively from the global north(27).	
Findings:<br>The sentence "Of this five, one patient allowed their relative to conduct the interview and creative task on their behalf..." seems unclear. Did the patient permit their relative to conduct the interview on their behalf, or was the relative simply participating? It would also help to clarify whether the authors were the ones conducting that particular interview or the participants.	Thank you for spotting that the use of conduct in this paragraph can cause ambiguity. We have reworded this to make it clear.<br><br>*Fifteen interviews were conducted with patients and or their relatives. Eight interviews were with patients alone, five patients also had relatives present, while two were with relatives only. Of the five interviews with patients and relatives, most involved joint participation of the patient with their relative, except, one patient who allowed their relative to solely partake in the interview and creative task on their behalf. On one occasion a relative stepped in to partake in the creative task only. The patient pool is summarised in Table 1.*	10
Discussion:<br>This section is well-written and supported by appropriate references.<br>However, could I suggest that the statement "Currently they have a small specialised palliative team (one nurse) serving the hospital." be reworded to something like ..."at the time of this study" This is just to clarify to readers and also enhance the credibility of the study as the team would have currently outgrown the "single nurse team" and anyone reading would appreciate this was only at the time of the study.	Thank you, very good point noted, we have updated as you suggest.<br><br>*At the time of this study, there was a small specialised palliative team (one nurse) serving the hospital.*	23
References to Fatalism:<br>The statement "Fatalism has been linked to cancer stigma (38). Fatalism was found to be a belief about cancer here and has as a longstanding tie to cancer, first articulated by Powe and Finne(44)." I think this statement could be further supported by citing a more relevant reference such as (https://pmc.ncbi.nlm.nih.gov/articles/PMC9040785/#sec17) which is directly related to the context and findings of this study. I think this could strengthen the connection to the current research context and add depth to the findings and discussion.	Thank you for sharing this. We fully agree and apologise for this omission as we only became aware of this publication submission but agree it adds further depth to the discussion.<br><br>*This is supported by other recent research that has suggested fatalism and distrust lead to cancer treatment refusal in Ghana(51). They suggest fatalism is linked to poor communications and a breakdown in negotiating the*	25

		patient pathway(51). This is in accordance with our findings, as opinions on and the support of health professionals influenced treatment behaviour. Moreover, the intersection between fatalism and feelings of futility in negotiating the pathway resonates with the concept of candidacy, used to better articulate the phenomena than 'access', by meaning along the full continuum of care(4,52).	

VERSION 2 - REVIEW

Reviewer 1

Name Okyere, Joshua

Affiliation University of Cape Coast, Department of Population and Health

Date 08-Feb-2025

COI

The authors have responded satisfactorily to the comments raised.
